# Does body mass index compromise assisted reproductive technique outcomes? A cross-sectional study

**DOI:** 10.18502/ijrm.v21i12.15040

**Published:** 2024-01-25

**Authors:** Fereshteh Bahrami, Saeideh Dashti, Esmat Mangoli, Hanie Sadat Hosseini

**Affiliations:** ^1^Research and Clinical Center for Infertility, Yazd Reproductive Sciences Institute, Shahid Sadoughi University of Medical Sciences, Yazd, Iran.; ^2^Department of Reproductive Biology, Research and Clinical Center for Infertility, Shahid Sadoughi University of Medical Sciences, Yazd, Iran.; ^3^Department of Physiology, School of Medicine, Shahid Sadoughi University of Medical Sciences, Yazd, Iran.

**Keywords:** Body mass index, Assisted reproductive technique, Pregnancy outcome, Live birth rate, Age.

## Abstract

**Background:**

Overweight and obese people face several health problems. Female obesity has been shown to reduce fertility in the general population. Assisted reproductive technology outcomes in obese cases are widely studied, but the results are inconclusive.

**Objective:**

This study aimed to compare live birth rate (LBR) among women with 4 different types of body mass index (BMI).

**Materials and Methods:**

In this cross-sectional study, data of 1611 women, who were candidates for fresh and frozen embryo transfer cycles, was extracted from 2051 medical files at the Reproductive Sciences Institute, Yazd, Iran from May 2019-May 2021. The participants were divided into 4 groups (underweight, normal, overweight, and obese) according to their BMI, and LBR was considered to be the main outcome.

**Results:**

Of 1611 women, 39 were underweight, 585 were normal, 676 were overweight, and 311 were obese. Underweight women had the lowest LBR (12.8%), but there was no statistically significant difference (p = 0.55). In addition, LBR was compared in the 4 BMI groups according to age, type of transfer cycle (fresh or freeze), and cause of infertility, and there was comparable LBR in the 4 BMI groups. However, metaphase 2 oocyte rate, doses of gonadotropin usage in the cycles, and estradiol level had statistically significant differences (p < 0.001).

**Conclusion:**

According to our study, obesity does not affect LBR in the IVF cycle, regardless of fresh or frozen embryo transfer cycles, different age groups, and causes of infertility.

## 1. Introduction

Lifestyle changes in modern societies have led to elevated body mass index (BMI) and obesity. In some countries, nearly 50% of the population is either overweight or obese (1). According to the World Health Organization (WHO), there are about 2 billion overweight and 650 million obese adults across the world. The issue is also common among people of reproductive age (2, 3). A systematic review showed that the prevalence of obesity and overweight in adults and children in Iran was about 12.8-76.4 and 2.4-35.4%, respectively (4). Also, a study in 2020 reported that around 59% of adults in Iran were overweight or obese (5).

Overweight and obese people face several health problems that are extensively documented. The negative effects of obesity on general health that are associated with the reproductive system are also remarkable. Research indicates that obesity in women can lead to reduced fertility and increase the risk of abortion in general population (6). However, similar conclusive results regarding the population requiring assisted reproductive technology (ART) have largely remained elusive. Some studies have linked obesity to diminished LBR, implantation rate, fertilization rate, and increased abortion rate due to decreased endometrial receptivity (7-10). Others have claimed that the oocytes of overweight and obese women are smaller than those of normal BMI women and have a faster growth rate after fertilization (11). Oocytes of overweight and obese cases have lower quantity and quality compared to those with healthy weight (12, 13). On the other hand, studies have found BMI to have no impact on the outcome of ART, and only obese women may need further doses of gonadotropins (14), and recommended reducing the BMI before ART is not necessary (15).

Given the inconclusiveness of the results reported in the literature, as well as the fact that all research in this field of data has been carried out in European and Northern American populations, this study aims to investigate the potential impacts of BMI on ART outcomes in both fresh and frozen cycles in Iranian population.

## 2. Materials and Methods

In this cross-sectional study, data of 1611 women (18-42 yr), who were candidates for fresh and frozen embryo transfer cycles, was extracted from a total of 2051 medical records at the Reproductive Sciences Institute, Yazd, Iran from May 2019-May 2021.

Women with uncontrolled underlying diseases, uterine anomaly, severe male factors like testicular samples or ejaculates with below 1 × 10^6^ spermatozoa/ml, uterine surrogacy, and ovum donation were excluded.

All relevant information, such as anti-Mullerian hormone (AMH) levels, BMI, cause and duration of infertility were all obtained from the participants' files. All women were categorized into 4 BMI groups according to the WHO classification: group 1 (underweight, BMI < 18.5 kg/m^2^), group 2 (normal, BMI = 18.5-24.9 kg/m^2^), group 3 (overweight, BMI = 25-29.9 kg/m^2^), and group 4 (obese, BMI ≥ 30 kg/m^2^). The protocol for ovarian stimulation was agonist or antagonist protocol, and the dosage of gonadotropin was adjusted according to the women's age, antral follicular count, and AMH levels.

In frozen embryo transfer cycles, endometrial preparation was done by estradiol valerate starting on the 2 ${\;}^{\text{nd}}$ day of the cycle, when endometrial thickness reached ≥ 7 mm, progesterone supplementation was administered. One or 2 embryos on day 3 were transferred on the 4 ${\;}^{\text{th}}$ day of progesterone administration.

IVF outcomes were compared between groups. The primary outcome was the live birth rate, and secondary outcomes were the implantation rate, abortion rate, and chemical and clinical pregnancy rate.

### Ethical considerations

All procedures comply with the ethical standards of the relevant national and institutional committees on human experimentation and with the Helsinki Declaration of 1975, as revised in 2008. This study was approved by the Ethical Committee of Reproductive Sciences Institute, Yazd, Iran (Code: IR.SSU.RSI.REC.1401.004).

### Statistical analysis

Statistical analysis was done by Statistical Package for the Social Sciences, version 18.0, SPSS Inc., Chicago, Illinois, USA (SPSS). Data processing was done by Chi-square and Fisher's exact tests in categorical variables. The distribution of continuous variables was checked by the Kolmogorov-Smirnov test, and the Kruskal-Wallis test was used for comparison in continuous variables. Subgroup analysis was performed by Chi-square or Fisher's exact test to assess LBR in different age groups and different embryo transfer cycles according to BMI. The significance level was considered as < 0.05.

## 3. Results

From a total of 1611 women who underwent ART treatment, 39 were underweight, 585 were normal, 676 were overweight, and 311 were declared obese. The mean age was statistically different between the 4 groups; however, this difference was not clinically significant.

Duration of infertility and serum levels of AMH were increased by an increment of BMI. However, this increase was not statistically significant. The total gonadotropin dose (p < 0.05) was found to be significantly different across the BMI categories, obese women needed further gonadotropin doses. The percentage of good quality embryos (grade A, B) and endometrial thickness (ET) did not differ between the groups. The percent of M2 oocytes was markedly lower in underweight women, and the difference between the 4 groups was statistically significant (p < 0.001). Also, the cause of infertility (p < 0.001), estradiol level (p < 0.001), and the number of transferred embryos (p < 0.001) had statistically significant differences between groups; the malefactor was the most common cause of infertility in underweight women. However, polycystic ovarian syndrome (PCOS) was the most common cause of infertility in other groups. Estradiol level decreased by an increment of BMI (Table I).

ART outcomes such as chemical and clinical pregnancy rate, live birth rate, and implantation rate did not differ significantly between the 4 groups. However, these outcomes had the lowest range in underweight women (Table II).

Subgroup analysis was done in 2 age groups ( < 35 yr, ≥ 35 yr) according to BMI (16). BMI had no significant effect on ART outcomes in each age group (Table III). Also, ART outcomes were assessed for different causes of infertility according to BMI, and the results were compared (Table IV). ART outcomes in fresh and frozen embryo transfer cycles did not differ in the 4 BMI groups separately (Table V). No significant difference was observed between LBR in fresh and frozen cycles. In normal BMI, LBR in the frozen embryo transfer group was higher than the fresh cycle (24.6% vs. 18.3%), and the difference was near to statistically significant, p = 0.07 (Table VI).

We considered that the baseline characteristics were statistically significant between the groups. Therefore, we considered confounding factors as categorical variables and investigated primary and secondary outcomes in subgroups. No difference in the subgroups of age was observed. We adjusted the confounding effect of age by logistic regression analysis and did not find a significant effect on outcomes.

**Table 1:** Basal and cycle characteristics of cases with different BMI


**Cases characteristic**		**Groups**				
		**Group 1 (n = 39)**	**Group 2 (n = 585)**	**Group 3 (n = 676)**	**Group 4 (n = 311)**	**P-value**
Age (yr)*		30.05 ± 5.64 (30.00, 7.00)	31.38 ± 5.06 (31.00, 7.00)	32.24 ± 5.18 (32.00, 7.00)	32.33.3 ± 5.12 (32.00, 8.00)		< 0.001
Infertility duration (yr)*		5.35 ± 3.16 (5.00, 4.00)	6.93 ± 4.01 (6.00, 5.00)	6.89 ± 4.16 (6.00, 5.00)	7.21 ± 4.32 (6.00, 6.00)	0.06
AMH (ng/ml)*		3.91 ± 3.47 (2.80, 3.00)	4.49 ± 3.66 (3.50, 3.90)	4.77 ± 4.08 (3.70, 5.20)	4.32 ± 3.89 (3.00, 4.00)	0.29
Cause of infertility**						
	**PCO**	7 (17.9)	174 (29.8)	244 (36.1)	99 (31.9)	
	**UI**	10 (25.6)	136 (23.2)	133 (19.7)	71 (22.8)	
	**DOR**	6 (15.4)	71 (12.1)	100 (14.8)	48 (15.4)	
	**Endometriosis**	2 (5.2)	15 (2.6)	17 (2.5)	5 (1.6)	
	**Male**	13 (33.3)	135 (23.1)	113 (16.7)	47 (15.1)	
	**Mixed**	1 (2.6)	54 (9.2)	69 (10.2)	41 (13.2)	< 0.001
Number of (transferred) embryos*		1.87 ± 0.33 (2.00, 0.00)	1.86 ± 0.34 (2.00, 0.00)	1.79 ± 0.40 (2.00, 0.00)	1.81 ± 0.39 (2.00, 0.00)		< 0.001
Gonadotropin dose (IU)*		1992.30 ± 706.66 (1650.00, 975.00)	2089.23 ± 911.47 (1800.00, 975.00)	2174.7 ± 917.88 (1875.00, 900.00)	2359.53 ± 903.02 (2100.00, 1050.00)		< 0.001
Good quality embryo**		37 (94.9)	548 (93.7)	620 (91.7)	277 (89.1)		0.09
M2 oocyte rate**		382/504 (75)	6012/7508 (80.1)	6364/7844 (81.1)	2516/3080 (81.7)		< 0.001
Endometrial thickness (mm)*		9.04 ± 1.46 (8.90, 2.00)	9.38 ± 1.84 (9.00, 2.00)	9.19 ± 1.77 (9.00, 2.00)	9.42 ± 1.78 (9.20, 2.00)		0.08
Estradiol (pg/ml)*		2446.30 ± 1931 (1700.00, 2560.00)	2273.37 ± 2042.43 (1650.00, 1736.00)	2079.35 ± 2142.54 (1430.00, 1497.00)	1699.94 ± 1719.76 (1202.00, 1190.00)	< 0.001
*Data are presented as Mean ± SD (Median, interquartile range), Chi-square test. **Data presented as n (%), Kruskal-Wall test. BMI: Body mass index, AMH: Anti-Mullerian hormone, PCO: Polycystic ovary syndrome, UI: Unexplained infertility, DOR: Diminished ovarian reserve, M2: Metaphase 2						

**Table 2:** Comparison of pregnancy outcomes according to BMI


	**Groups**				
**Variables**	**Group 1 (n = 39)**	**Group 2 (n = 585)**	**Group 3 (n = 676)**	**Group 4 (n = 311)**	**P-value**
	**Chemical pregnancy**	7 (17.9)	179 (30.6)	216 (32)		92 (29.6)	0.30
**Clinical pregnancy**	6 (15.4)	141 (24.1)	168 (24.9)	69 (22.2)	0.49
**Ongoing pregnancy**	5 (12.8)	132 (23.5)	159 (23.5)	66 (21.6)	0.42
**Live birth**	5 (12.8)	127 (22.3)	151 (22.3)	65 (20.9)	0.55
**Implantation rate**	6/73 (8.2)	158/1092 (14.5)	180/1215 (14.8)	89/564 (15.8)	0.38
**Abortion rate**	1/6 (16.7)	8/141 (5.7)	16/168 (9.5)	4/69 (5.8)	0.32
Data are presented as n (%), the Chi-square test. BMI: Body mass index					

**Table 3:** Pregnancy outcomes in each age group according to BMI


**Age (yr)**		**Groups**				
		**Group 1 (n = 39)**	**Group 2 (n = 585)**	**Group 3 (n = 676)**	**Group 4 (n = 311)**	**P-value**
** < 35 (n = 1098)**						
	**Chemical pregnancy**	7/32 (21.9)	149/431 (34.6)	159/434 (36.6)	67/201 (33.3)	0.36
	**Clinical pregnancy**	6/32 (18.8)	121/431 (28.1)	124/434 (28.6)	52/201 (25.9)	0.61
	**Ongoing pregnancy**	5/32 (15.6)	114/431 (26.5)	119/434 (27.4)	49/201 (24.4)	0.46
	**Live birth rate**	5/32 (15.6)	109/431 (25.3)	114/434 (26.3)	48/201 (23.9)	0.57
	**Abortion rate**	1/7 (14.3)	8/149 (5.4)	9/159 (5.7)	4/67 (6)	0.60
	**Implantation rate**	6/61 (9.8)	135//809 (16.7)	134/798 (16.8)	63/368 (17.1)	0.54
** ≥ 35 (n = 513)**						
	**Chemical pregnancy**	0/7 (0)	30/154 (19.5)	57/242 (23.6)	25/110 (22.7)	0.40
	**Clinical pregnancy**	0/7 (0)	20/154 (13)	44/242 (18.2)	17/110 (15.5)	0.35
	**Ongoing pregnancy**	0/7 (0)	18/154 (11.7)	40/242 (16.5)	17/110 (15.5)	0.38
	**Live birth rate**	0/7 (0)	18/154 (11.7)	37/242 (15.3)	17/110 (15.5)	0.50
	**Abortion rate**	- 1/30 (3.3)	7/57 (12.3)	0/25 (0)	0.10
	**Implantation rate**	0/12 (0)	23/283 (8.1)	46/417 (11)	26/196 (13.3)	0.18
Data are presented as n (%), the Chi-square test. BMI: Body mass index						

**Table 4:** Pregnancy outcomes of each infertility cause according to BMI

**Cause of infertility**	**Groups**	**P-value**
	**Group 1 (n = 39)**	**Group 2 (n = 585)**	**Group 3 (n = 676)**	**Group 4 (n = 311)**	
**PCO (n = 542)**
	**Chemical pregnancy**	1/7 (14.3)	66/174 (37.9)	76/244 (31.1)	29/99 (29.3)	0.29
	**Clinical pregnancy**	1/7 (14.3)	55/174 (31.6)	59/244 (24.2)	21/99 (21.2)	0.17
	**Ongoing pregnancy**	1/7 (14.3)	50/174 (28.7)	57/244 (23.4)	20/99 (20.2)	0.35
	**Live birth rate**	1/7 (14.3)	46/174 (26.4)	56/244 (23)	18/99 (18.2)	0.43
	**Abortion rate**	0/1 (0)	5/66 (7.6)	5/76 (6.6)	3/29 (10.3)	0.81
	**Implantation rate**	1/14 (7.1)	56/338 (16.6)	64/459 (13.9)	28/184 (15.2)	0.62
**Unexplained (n = 350)**
	**Chemical pregnancy**	1/10 (10)	39/136 (28.7)	46/133 (34.6)	20/71 (28.2)	0.33
	**Clinical pregnancy**	1/10 (10)	32/136 (23.5)	36/133 (27.1)	16/71 (22.5)	0.60
	**Ongoing pregnancy**	1/10 (10)	32/136 (23.5)	33/133 (24.8)	14/71 (19.7)	0.64
	**Live birth rate**	1/10 (10)	32/136 (23.5)	32/133 (24.1)	15/71 (21.1)	0.75
	**Abortion rate**	0/1 (0)	0/39 (0)	3/46 (6.5)	1/20 (5)	0.27
	**Implantation rate**	1/19 (5.3)	39/254 (15.4)	42/264 (15.9)	22/133 (16.5)	0.64
**DOR (n = 225)**
	**Chemical pregnancy**	0/6 (0)	17/71 (23.9)	28/100 (28)	13/48 (27.1)	0.57
	**Clinical pregnancy**	0/6 (0)	9/17 (12.7)	23/100 (23)	9/48 (18.85)	0.28
	**Ongoing pregnancy**	0/6 (0)	9/71 (12.7)	21/100 (21)	9/48 (18.8)	0.41
	**Live birth rate**	0/6 (0)	9/71 (12.7)	19/100 (19)	9/48 (18.8)	0.46
	**Abortion rate**	-	1/17 (5.9)	3/28 (10.7)	0/13 (0)	0.80
	**Implantation rate**	0/9 (0)	10/123 (8.1)	23/148 (15.5)	11/85 (12.9)	0.18
**Endometriosis (n = 39)**
	**Chemical pregnancy**	1/2 (50)	5/15 (33.3)	2/17 (11.8)	1/5 (20)	0.31
	**Clinical pregnancy**	1/2 (50)	3/15 (20)	0/17 (0)	1/5 (20)	0.05
	**Ongoing pregnancy**	1/2 (50)	3/15 (20)	0/17 (0)	1/5 (20)	0.05
	**Live birth rate**	1/2 (50)	3/15 (20)	0/17 (0)	1/5 (20)	0.05
	**Abortion rate**	0/1 (0)	-	-	-	-
	**Implantation rate**	1/4 (25)	3/27 (11.1)	0/31 (0)	1/0 (11.1)	0.15
**Male factor (n = 308)**
	**Chemical pregnancy**	3/13 (23.1)	37/135 (27.4)	34/113 (30.1)	15/47 (31.9)	0.88
	**Clinical pregnancy**	2/13 (15.4)	29/135 (21.5)	28/113 (24.8)	12/47 (25.5)	0.80
	**Ongoing pregnancy**	1/13 (7.7)	25/135 (18.5)	27/113 (23.9)	12/47 (25.5)	0.38
	**Live birth rate**	1/13 (7.7)	24/135 (17.8)	26/113 (23)	12/47 (25.5)	0.37
	**Abortion rate**	1/3 (33.3)	3/37 (8.1)	2/34 (5.9)	0/15 (0)	0.27
	**Implantation rate**	2/25 (8)	33/252 (13.1)	30/202 (14.9)	15/82 (18.2)	0.52
**Mixed (n = 165)**
	**Chemical pregnancy**	1/1 (100)	15/54 (27.8)	30/69 (43.5)	14/41 (34.1)	0.14
	**Clinical pregnancy**	1/1 (100)	13/54 (24.1)	21/69 (31.9)	10/41 (24.4)	0.33
	**Ongoing pregnancy**	1/1 (100)	13/54 (24.1)	21/69 (30.4)	10/41 (24.4)	0.37
	**Live birth rate**	1/1 (100)	13/54 (24.1)	18/69 (26.1)	10/41 (24.4)	0.49
	**Abortion rate**	0/1 (0)	0/15 (0)	3/30 (10)	0/14 (0)	0.44
	**Implantation rate**	1/2 (50)	17/98 (17.3)	21/119 (17.6)	12/71 (16.9)	0.68

Data are presented as n (%), the Chi-square test. BMI: Body mass index, PCO: Polycystic ovary, DOR: Diminished ovarian reserve

**Table 5:** Pregnancy outcomes in fresh/freeze cycles according to BMI


**Cycle **		**Groups**				
		**Group 1 (n = 39)**	**Group 2 (n = 585)**	**Group 3 (n = 676)**	**Group 4 (n = 311)**	**P-value**
**Freeze (n = 785)**						
	**Chemical pregnancy**	4/20 (20)	107/317 (33.8)	114/328 (34.8)	36/120 (30)	0.47
	**Clinical pregnancy**	3/20 (15)	89/317 (28.1)	86/328 (26.2)	25/120 (20.8)	0.30
	**Ongoing pregnancy**	3/20 (15)	83/317 (26.3)	82/328 (25)	24/120 (20)	0.42
	**Live birth rate**	3/20 (15)	78/317 (24.6)	79/328 (24.1)	24/120 (20)	0.59
	**Abortion rate**	0/4 (0)	7/107 (6.5)	6/114 (5.3)	1/36 (2.8)	0.84
	**Implantation rate**	3/38 (7.8)	97/611 (15.8)	91/615 (14.7)	31/222 (13.9)	0.72
**Fresh (n = 826)**						
	**Chemical pregnancy**	3/19 (15.8)	72/268 (29.3)	102/348 (29.3)	56/191 (29.3)	0.57
	**Clinical pregnancy**	3/19 (15.8)	52/268 (19.4)	82/348 (23.6)	44/191 (23)	0.55
	**Ongoing pregnancy**	2/19 (10.5)	49/268 (18.3)	77/348 (22.1)	42/191 (22)	0.42
	**Live birth rate**	2/19 (10.5)	49/268 (18.3)	72/348 (20.7)	41/191 (21.5)	0.58
	**Abortion rate**	1/3 (33.3)	2/72 (2.8)	10/102 (9.8)	3/56 (5.4)	0.07
	**Implantation rate**	3/35 (8.5)	61/481 (12.6)	89/600 (14.8)	58/342 (16.9)	0.72
Data are presented as n (%), the Chi-square test. BMI: Body mass index						

**Table 6:** Pregnancy outcome (live birth rate) in BMI groups according to fresh and frozen cycle


**Age**	**Frozen cycle (n = 785)**	**Fresh cycle (n = 826)**	**P-value**
**Underweight**	3/20 (15)	2/19 (10.5)	1.00
**Normal**	78/317 (24.6)	49/268 (18.3)	0.07
**Overweight**	79/328 (24.1)	72/348 (20.7)	0.31
**Obese**	24/120 (20)	41/191 (21.5)	0.77
Data are presented as n (%), the Chi-square test. BMI: Body mass index			

## 4. Discussion

A total of 1611 women were grouped based on their BMI, and 42% of the majority of the subjects were in the overweight group. The main objective of this study was to compare LBR among women with 4 different types of BMI. The outcomes were compared among the underweight group which had the lowest LBR. However, due to the small number of cases in this group (only 2.5% of the entire study population), a significant p-value was not observed. In the literature, we found increased BMI to impact IVF. In a retrospective cohort study in 2020 in China on 14,782 cycles, the correlation of BMI and cumulative live birth rate (CLBR) was found to be an inverted. Specifically, CLBR was highest in underweight, plateaued in normal and overweight, and decreased in obese women. However, the BMI group's definition was not according to the WHO (17). In another retrospective cohort study on 2,39,127 fresh embryo transfers, which was published in 2016, LBR decreased progressively with increasing BMI (8). These results were confirmed by other studies (7, 18).

Contrary to these studies, in 2020, in a retrospective cohort of 1415 cases of blast transfer, the LBR in obese people who had frozen blast embryo transfer was examined and was found to be the same as normal people. Therefore, it was concluded that obesity has no harmful effect on endometrial receptivity (3). Moreover, in a retrospective cohort study in 2018, obesity was not found to have a negative effect on the cumulative pregnancy rate. This study was conducted on 1345 cycles of single embryo transfer; and 292 people in this study had high BMI, and 864 people had normal BMI (1). In 2016, in a retrospective study, 1602 cases for which the first fresh transfer was performed were divided into 2 groups, obese and normal, and no difference was found in LBR, but the rate of abortion increased with an increase in BMI. Also, no significant difference in the number and quality of oocytes and embryos were observed. This study was conducted in an Italian population (19), and the results were different from earlier studies in the North American populations, which authors attributed to the different genetic and lifestyle factors.

In this study, doses of gonadotropin were significantly higher in obese cases, which is similar to the result of a previous study (20). Although the requirement for higher doses of gonadotropin in obese and overweight cases is logical, another study found no correlation between BMI and doses of gonadotropin (21).

Regarding the impact of BMI on M2 oocytes, some have concluded that high BMI reduces M2 oocyte count (22), while others have reported contradictory results, indicating no impact from higher BMI (23). Our results suggest a significant reduction in M2 oocytes in the underweight group. Nevertheless, in agreement with other reports in the literature, this reduction in M2 oocytes did not affect good-quality embryo numbers (6, 24). Therefore, the reduced M2 oocyte count is not clinically important. Yet, there are studies such as that believe in a reduction of good-quality embryos with increased BMI (25).

Endometrial thickness (ET) is another important factor in successful embryo transfer (26, 27). In this study, ET was compared among 4 BMI groups. It should be noted that other studies have suggested a significant difference in ET according to BMI (3, 28). However, even in these studies, the ET variation with the BMI was less than 1 mm, and such minor differences do not appear to have any clinical importance. A study assessed the relationship between BMI and AMH and found that AMH level increases in obese cases (29). The result was similar to a study, where AMH level was comparable in the 4 BMI groups (30). Since the source of AMH is preantral and small antral follicles, which are not impacted by obesity, it is reasonable that obesity has no impact on AMH levels.

We analyzed our results based on the BMI in subgroups of age and found no significant impact of BMI on IVF outcomes in both < 35 and > 35 yr of age. Moreover, we found that higher BMI does not impact IVF outcomes regardless of the underlying cause of infertility. Only in PCO, women with normal BMI had an 8% higher LBR compared to obese subjects. This reduced LBR in obese cases is likely due to metabolic changes in obese PCO women.

In all the previous studies, most analyses focused on fresh cycles, with a few studies on freeze cycles. However, in the present study, we analyzed both fresh and freeze cycles. Pregnancy results among the 4 BMI groups were compared, and in comparing fresh and freeze cycles regarding the LBRs, it was found that in the normal BMI group, the freeze cycle was more effective with close to a significant p-value of 0.07. However, for the obese group, no meaningful difference was found between the fresh and freeze cycles.

It seems that the main discrepancy in our result with studies is the genetic diversity of our population and perhaps the larger sample size of those works (8, 17). Thus, we recommend a large multicentric study about this context in our country. The main limitation of our study is its retrospective nature, leading to some missing data points in the participants' files. As such, a number of the patient files were removed from the study due to missing required information. However, the analysis of multiple aspects of ART cycles, only a few of which had been considered in earlier studies, may cover the main shortcomings of the work.

## 5. Conclusion

According to our findings and some similar studies, BMI may not be a prognostic factor for ART outcomes. Factors such as age, antral follicular count, and AMH are the best prognostic factors. It is recommended to conduct a multi-center prospective study with a larger sample size and the impact of some factors like lifestyle, previous ART cycles, or comorbidity.

##  Conflict of Interest

The authors declare that there is no conflict of interest.
